# A New Spin on Research Translation: The Boston Consensus Conference on Human Biomonitoring

**DOI:** 10.1289/ehp.0800037

**Published:** 2008-10-30

**Authors:** Jessica W. Nelson, Madeleine Kangsen Scammell, Rebecca Gasior Altman, Thomas F. Webster, David M. Ozonoff

**Affiliations:** 1 Department of Environmental Health, Boston University School of Public Health, Boston, Massachusetts, USA; 2 Department of Sociology, Brown University, Providence, Rhode Island, USA

**Keywords:** biomonitoring, consensus conference, environmental health surveillance, participatory democracy, research translation

## Abstract

**Background:**

Translating research to make it more understandable and effective (research translation) has been declared a priority in environmental health but does not always include communication to the public or residents of communities affected by environmental hazards. Their unique perspectives are also commonly missing from discussions about science and technology policy. The consensus conference process, developed in Denmark, offers a way to address this gap.

**Objectives:**

The Boston Consensus Conference on Human Biomonitoring, held in Boston, Massachusetts, in the fall of 2006, was designed to educate and elicit input from 15 Boston-area residents on the scientifically complex topic of human biomonitoring for environmental chemicals. This lay panel considered the many ethical, legal, and scientific issues surrounding biomonitoring and prepared a report expressing their views.

**Discussion:**

The lay panel’s findings provide a distinct and important voice on the expanding use of biomonitoring. In some cases, such as a call for opt-in reporting of biomonitoring results to study participants, they mirror recommendations raised elsewhere. Other conclusions have not been heard previously, including the recommendation that an individual’s results should be statutorily exempted from the medical record unless permission is granted, and the opportunity to use biomonitoring data to stimulate green chemistry.

**Conclusion:**

The consensus conference model addresses both aspects of a broader conception of research translation: engaging the public in scientific questions, and bringing their unique perspectives to bear on public health research, practice, and policy. In this specific application, a lay panel’s recommendations on biomonitoring surveillance, communication, and ethics have practical implications for the conduct of biomonitoring studies and surveillance programs.

Improving “research translation” has become a top priority of government agencies. In 2003, the National Institute of Environmental Health Sciences (NIEHS) formalized research translation with the requirement of Community Outreach and Translation Cores at each of the Centers for Children’s Environmental Health and Disease Prevention Research. The NIEHS defines translation as the process of using basic research to inform “intervention and prevention methods to enhance awareness among communities, health care professionals, and policy makers of environmentally related diseases and health conditions” (NIEHS Division of Extramural Research and Training 2006). It has become clear that effective research translation involves collaboration among scientists, government officials, and individuals or communities affected by environmental issues.

The phrase “research translation” is ambiguous and often is used loosely to refer to the communication of research findings to professional audiences outside university or government research laboratories, such as risk assessors, pharmaceutical companies, and congressional aides, rather than to lay communities facing environmental problems. In some cases, general research translation has displaced direct community outreach. For example, although the NIEHS Superfund Basic Research Program (SBRP) once encouraged a Community Outreach Core, this remains optional for program applications, whereas the Research Translation Core is mandatory.

We describe the consensus conference model as one strategy for translating basic research in a way that overcomes social and technical barriers and thus allows us to include lay communities in the translation mix. The consensus conference process was used to educate and elicit input from members of the lay public on the scientifically complex topic of human biomonitoring for environmental chemicals. In this commentary we highlight what the process entails and report the findings our consensus conference lay panel released on human biomonitoring programs and their subsequent use by policy makers. The consensus conference project is a component of the Research Translation Core of the Boston University (BU) SBRP. The core carries out programs to systematically reduce barriers to effective research translation. With this project, we adopt a view of research translation that includes translating basic research to end users and perspectives from the lay public (beyond the bench) back into public health and research practice, a process we refer to as “simultaneous research translation.”

## Background

Human biomonitoring, the practice of measuring chemicals in human tissues and fluids, has advanced dramatically over the last decade (Stokstad 2004; Suk et al. 1996). Clinicians, researchers, government agencies, and environmental health advocacy groups now employ the technology for a variety of purposes. For example, the Centers for Disease Control and Prevention (CDC) conducts routine biomonitoring surveillance of the population through the National Health and Nutrition Examination Survey (NHANES) program. States are increasingly considering biomonitoring surveillance programs of their own. California passed legislation establishing the first of these in September 2006, and Minnesota followed in May 2007. Indiana, New York, and Washington State also have introduced similar legislation.

Biomonitoring is a topic that has sparked some controversy, including concerns about the interpretation of biomonitoring results and whether and how such results should be communicated to the public and to research participants (Brody et al. 2007; Paustenbach and Galbraith 2006; Schmidt 2006). In 2006, the National Research Council (NRC) published a report representing 2 years of deliberation by a panel of experts on the scientific and ethical challenges of human biomonitoring (NRC 2006). Industry has also sponsored similar panels (Angerer et al. 2006; Bahadori et al. 2007; Bates et al. 2005). The voice of the public, however, has been absent from these discussions. To begin to fill this gap, the BU School of Public Health (SPH) convened a Danish-style consensus conference on biomonitoring in fall 2006.

In Denmark, consensus conferences are used to stimulate social debate and inform policy making on emerging science and technology issues. The process involves recruiting a “lay panel” of residents, similar to jury duty in the United States. Their views are not meant to be representative of the lay public, but rather to highlight themes and concerns that exist among members of the general public. The Danish government has convened consensus conferences on topics as varied as the regulation of genetically modified food, the use of new knowledge about the human brain, and electronic surveillance—topics that address a current controversy, require experts to clarify technical and scientific aspects, and are related to pending legislation or policy (Grundahl 1995; Steyaert and Lisor 2005).

The Boston Consensus Conference on Human Biomonitoring assembled 15 lay people from the Boston area to consider ethical, legal, social, and scientific issues related to the practice of measuring chemicals in peoples’ bodies. We followed the Danish model, as described by Grundahl (1995), as closely as possible, while recognizing that the approach has been further developed over time. In addition to funding from the BU SBRP, the project was supported by an Environmental Justice grant awarded to BU SPH by NIEHS to provide education on the ethical issues and challenges related to conducting environmental health studies and to establish dialogue between scientists and community members.

Here we summarize the process of convening the lay panel, present the panel’s findings, and discuss how these findings have been received by policy makers, public health scientists, and agencies. In particular, we highlight the unique contributions made by the lay panel to current thinking regarding government biomonitoring surveillance programs, communication about biomonitoring study results, and ethics of right to know, privacy, and confidentiality.

## Consensus Conference: Process and Preparation

The Boston Consensus Conference on Human Biomonitoring involved three weekend meetings from October through December 2006. Over the first two weekends, the lay panel learned about biomonitoring through a facilitated curriculum of reading, expert testimony, and discussion. As they gained knowledge on the subject, the panelists began to identify and articulate key questions and concerns. During a third weekend, they posed these questions to a panel of experts and then summarized their findings and recommendations in a written Consensus Statement, which was presented in a public meeting.

The project was overseen by project staff at BU SPH, a team of professional facilitators with experience facilitating a prior consensus conference (Sclove 1997), and a steering committee [see Supplemental Material A (http://www.ehponline.org/members/2008/0800037/suppl.pdf)], which included seven experts from the fields of academia, government, industry, and advocacy. The steering committee’s role was to provide balanced oversight of the project and a diversity of perspectives on the topic.

### Assembling the lay panel

The 15 panelists reflected the demographics of the City of Boston in terms of age, sex, race/ethnicity, and income according to 2000 census data. Our goal of a 15-member panel was small enough to reach meaningful consensus in a limited time frame but large enough to allow for losing members to unforeseeable circumstances while maintaining a functional, diverse group. Occupation, education, and whether or not participants had children were also considered when selecting participants, who were recruited by placing ads in local newspapers and on Craigslist, posting fliers, and distributing postcards throughout the city. Everyone who responded with interest was contacted by phone and went through a preliminary screening. Those who remained interested and available on meeting dates were sent a questionnaire, which 65 people completed and returned. Participants were selected from this group to satisfy demographic balance, while also ensuring that none had prior experience with biomonitoring. The final panel [see Supplemental Material B (available online at http://www.ehponline.org/members/2008/0800037/suppl.pdf)] was a diverse group from various Boston neighborhoods and surrounding communities. Three participants had children < 13 years of age. Occupations included, among others, truck driver, attorney, youth detention center employee, and manager in a pharmaceutical company. Panel members who attended all sessions were equally compensated for their time and effort.

### Educational materials and presentations

Members of the lay panel were initially given a briefing paper, selected readings, and presentations by experts in the field [Supplemental Material, Appendix A (available online at http://www.ehponline.org/members/2008/0800037/suppl.pdf)]; additional information is available from the project Web site, www.biomonitoring06. org). The first weekend, two BU SPH scientists gave the panel a general overview of environmental health and the science of biomonitoring. The lay panel’s own questions and concerns determined the content of educational materials and presentations used for subsequent weekends. During the second weekend (3 weeks later), as questions from the lay panel became more focused, they heard from experts from both the national CDC surveillance program and the newly formed California surveillance program.

### Deliberations

During the first weekend, the professional facilitators worked to establish trust and a commitment to the process among panel members and facilitators. The group agreed on a set of ground rules for communication and spent several hours defining the meaning of consensus: to reach consensus, all panel members needed to feel they could live with the decision and that their concerns had been voiced and heard by the group. Each time the facilitators presented a decision to the group, panel members closed their eyes and indicated their level of agreement by holding up one to four fingers, one indicating enthusiastic support and four disagreement and blocking of consensus. From the outset the lay panel began to articulate their key questions about biomonitoring. Working in small groups with the facilitators, panelists wrote questions and comments on index cards, tacked them to boards, arranged them by subject, and discussed them. At the end of the weekend, the facilitators organized the panel’s lengthy list of issues into 10 categories, for example, “ethics—confidentiality and disclosure” and “outreach, access, and control.”

During the second weekend, the lay panel further refined their questions and concerns about biomonitoring. After reviewing, adding to, and ranking issues identified the first weekend, the group reached consensus on the key areas they wanted to consider. Using this new framework, small groups worked to delve more deeply into the issues and to clarify remaining questions. At the end of this process, the conference room walls were covered with cards and poster boards. All were transcribed into a document, and the facilitators consolidated this into what would become the framework for the lay panel’s Consensus Statement.

Remaining questions from the second weekend informed selection of an expert panel for the third and final weekend meeting. With the help of the steering committee, project staff identified experts who could address the panels’ most pressing questions. Despite the short notice, expert participation was generous and enthusiastic. The expert panel took place on the Saturday of the final weekend [see Supplemental Material, Appendix A (available online at http://www.ehponline.org/members/2008/0800037/suppl.pdf)]. Each expert gave a 20-min presentation that responded to questions of the lay panel, and presentations were followed by open discussion. Lay panelists posed questions directly to the experts, or wrote them on cards that were read aloud by project staff. Members of the public in attendance were invited to ask questions once the lay panel finished asking their own.

After public discussion, the panelists were given time to take notes and reflect on the information. At the end of the day, the lay panel and facilitators met privately to review and discuss how the new information fit with their summary document from the second weekend. They added new opinions or ideas and inserted comments for group discussion. The facilitators then compiled all comments into a single document in preparation for the final working session.

The next day, the group finalized the Consensus Statement by reviewing the draft document in detail. They discussed each statement one by one and worked to reach consensus. In a few cases, the group could not reach consensus on the inclusion of a particular point or phrasing and agreed to exclude it from the final document. Each section of the statement was written by participants through a facilitated group process, with editing of the larger document by facilitators and project staff, and final review by the lay panel.

On the last day, the lay panel presented their findings at a public event. Panelists read from the Consensus Statement and answered questions from the audience in a lively session. Those in attendance included representatives from legislative offices and public health agencies (the offices of Massachusetts state senators, the newly elected governor’s transition team working group on energy and the environment, the Boston Public Health Commission, and the New Hampshire Department of Health and Human Services), academic researchers and students of environmental health, community-based and environmental advocacy organizations, a chemical industry trade association, and members of the press. The Consensus Statement and a 17-min video on the project produced by BU SPH are available on the project’s Web site (www.biomonitoring06.org).

## Findings: Lay Panel Consensus Statement on Human Biomonitoring

In the preamble to the Consensus Statement, the panel recognizes biomonitoring as an important public issue that “hits close to home” for many of them. The statement identifies and makes recommendations on four areas the panel feels need further consideration as the practice of biomonitoring moves forward: *a*) establishing responsible surveillance programs; *b*) using biomonitoring data to influence corporate and government behavior; *c*) educating the general public about biomonitoring; and *d*) addressing the issues of ethics, confidentiality, and disclosure.

### Establishing responsible surveillance programs

The Consensus Statement supports the need for biomonitoring surveillance:

By providing the ongoing, systematic collection, analysis, interpretation, and dissemination of biomonitoring data, surveillance programs demonstrate to both the scientific community and the public that [chemicals are present in human bodies] and potentially provide real information for public health intervention.

The panel hopes biomonitoring programs will encourage the allocation of more funds for studies on the health effects of exposure to chemicals.

The panel offers two noteworthy recommendations. First, the panel recommends that biomonitoring surveillance oversight boards be composed of different stakeholder groups, including individuals from affected communities. As an example, the panel cites the advisory council for the Massachusetts lead surveillance program, which includes parents of children who live in low-income communities and may be adversely affected by lead exposure. Second, the panel believes that state-based biomonitoring surveillance programs are a useful adjunct to the federal program. They argue that state programs may capture unique regional and local exposure patterns, and that because states are often the driving force behind important public health measures, state-specific information enables more informed local decision making and consumer choices.

### Using biomonitoring data to influence corporate and government behavior

Throughout their deliberations, some members of the group expressed concern about accountability for chemical pollution, citing examples of corporate and government culpability for improperly managed hazardous waste sites and other sources of chemical exposures. Many shared the desire to have biomonitoring data influence corporate and government behavior, particularly by stimulating the development of “green chemistry” and “green production”:

Our hope is that biomonitoring, by helping us understand which chemicals are increasing in our populations and guiding research on health outcomes, will lead to greater accountability and responsibility on the part of industry, … to more consistent compliance with regulations, and to advances in public health and medicine.

The panel considered how biomonitoring results may highlight a need to address exposure reduction, even in the face of uncertain health implications:

Biomonitoring data showing an increasing trend in exposure to a chemical, even when the health effects are uncertain, should be treated in a precautionary manner that seeks to reduce or eliminate exposure.

### Educating the general public about biomonitoring

The panel felt that educating the general public about biomonitoring was essential for achieving broad participation in biomonitoring surveillance programs and research studies. They identified biomonitoring as a portal into the health care system, especially for communities who have historically lacked both access to and trust in health care providers:

The experience of people in a biomonitoring program can be both an opportunity and a risk for their attitude and trust in a health services system that has not always served all members of our community equally.

The panel also recommended that biomonitoring information be shared with the public in broad, accessible public education programs. The panel is aware that biomonitoring results may be misconstrued and that two groups may interpret the same information differently, depending on their own interests and backgrounds:

The information taught or communicated has to be precise, which includes conveying accurate information about what is known and not known about cause and effect of exposure to monitored chemicals. This can be difficult to do and take time; however, it’s an essential part of educating the general public in a way that does not raise inappropriate alarm.

### Addressing the issues of ethics, confidentiality, and disclosure

Concern about how individuals could be harmed by biomonitoring results was the issue about which panel members had the strongest feelings. Although they were instructed about institutional review board requirements and mandated measures to protect human research subjects, they remained concerned about the potential for biomonitoring results to inadvertently or otherwise become attached to a person’s medical record without full consideration of the consequences for insurance or employment status. Among the ramifications noted in the Consensus Statement is the potential for the discovery of new information about a chemical’s toxicity long after a sample has been analyzed, which may render a once insignificant finding legally or medically significant. Importantly, panelists recommend that biomonitoring data be treated as a protected class of medical information:

It is the consensus of the panel that information derived from biomonitoring, as with that from genetic testing and AIDS results reporting, should be statutorily exempted from being transmitted or shared with employers, insurers or others as part of the medical history, without the express written consent of the individual. Specifically, it is recommended that legislation be enacted to ensure this.

Panelists also underscore that confidentiality is critical to ensuring public trust in biomonitoring programs, and that it is imperative that scientists and government officials consider—and better address—the unique confidentiality issues raised by biomonitoring.

Finally, the group asserts that study participants should be able to decide whether or not they want to receive their personal results, and that an important element of this report be inclusion of action steps for reducing exposure, when these are available.

## Discussion

Thomas Burke (Risk Sciences and Public Policy Institute at John Hopkins Bloomberg School of Public Health, and chair of the NRC Biomonitoring Committee) said the lay panel clearly understood the issue and had moved biomonitoring forward. The Consensus Conference elicited lay people’s unique insights on biomonitoring that may be essential to the future development of biomonitoring practice and policy. The project and its findings have been presented at numerous conferences and meetings, including those of the American Public Health Association, International Society for Environmental Epidemiology, Massachusetts Public Health Association, Minnesota Environmental Health Tracking and Biomonitoring Advisory Panel, the U.S. Environmental Protection Agency, and International Council of Chemical Associations. Articles have been published in a variety of forums, including trade and industry press (Dunn 2008; Nelson 2007a, 2007b; Rizzuto 2007a, 2007b).

Some of the panel’s concerns reflect issues raised in other venues, such as the NRC report (NRC 2006) and deliberations about the California legislation. These include recommendations for allowing participants to choose whether or not they want to receive their biomonitoring results; increased public education on biomonitoring that is done in an objective, meaningful manner and that takes into account different educational and cultural backgrounds; and the establishment of surveillance program oversight boards that include non-expert community members. Although these issues are not new, expert opinion has been divided, particularly on the question of reporting back results to biomonitoring participants. The view of the lay public is another “data point” in these deliberations. For example, John Dreisig, a toxicologist with New Hampshire’s Public Health Laboratories, attended the lay panel’s presentation of their findings. New Hampshire has been working on biomonitoring projects over the past several years to assess exposure to environmental contaminants such as arsenic and mercury, with grant funding from the CDC. Dreisig said the lay panel’s consensus on the importance of reporting biomonitoring results to study participants affirmed his own program’s commitment to communicating individual results back to participants in their arsenic biomonitoring study. The Consensus Statement supported investing resources in the communication of findings and general education of the public on study results (Dreisig J, personal communication).

The panel also provided recommendations and insights that have not, to our knowledge, been voiced in the literature or by expert panels. Notable among them is the recommendation that an individual’s biomonitoring results be statutorily exempted from their medical record unless express permission is granted. The concern about confidentiality was significant for the lay panel in ways that would affect participation in biomonitoring programs/studies.

The lay panel found that effective translation of biomonitoring results should involve carryover to corporate and government practices. From the beginning of their deliberations, some vocal members of the panel were action-oriented and apt to raise solutions to environmental health problems without necessarily considering their relevance to biomonitoring. After being challenged by the facilitators to connect the issue of corporate and government accountability to the topic at hand, they articulated a conclusion about the potential for biomonitoring data to stimulate green chemistry, a view that is both novel and important. The panel concluded that upward trends in biomonitored compounds should trigger precautionary action even if health effects are uncertain. Although these concerns may be considered tangential by some professionals, they are central to the purpose of biomonitoring for some lay panelists.

Other public health officials have also expressed to us the value of eliciting an informed lay perspective on these questions. In California, the first state to pass biomonitoring surveillance legislation, the Consensus Statement has been used as a tool for educating lay community members at workshops on designing the new state program. According to Amy Kyle (University of California-Berkeley School of Public Health, personal communication),

The Consensus Statement is written in a different language than that used by scientists. It is accessible to a different audience and is more readable and multidimensional than many other documents about biomonitoring.

The Consensus Conference also illuminated the potential of the Danish model to involve the public in complex science and technology policy discussions. Each lay panelist brought his or her own background to the discussions of biomonitoring, and most had no prior knowledge about the technology. The lay panel’s findings offer a distinct and important viewpoint to add to those of academia, government, and industry. As one member of the lay panel noted, “This is a good way to include the voices of ‘average folks’ and their uniquely relevant experiences in the policy making and public education process.” The strength of using a consensus-based model was also apparent. Coming to agreement on particular issues forced panel members to delve into the assumptions underlying their opinions, and to justify and question them in a way that does not happen in a majority-rules decision.

Dorothy Sussman (Division of Laboratory Sciences, National Center for Environmental Health, CDC) said the lay panel’s Consensus Statement reinforced the importance and value of public health agencies getting public input. CDC has since issued a communications research contract on public perceptions of biomonitoring. The Consensus Statement is also used by Sussman in her public presentations on the challenges of communicating biomonitoring surveillance results to the public (Sussman D, personal communication).

As is the case with many valuable projects, this was a labor- and resource-intensive effort. Facilitation, staffing, and compensation of the lay panel for their time made up the bulk of the roughly $75,000 budget. Even with the advantage of insights from the project team involved with an earlier consensus conference, the learning curve was steep for the organizers. Some of these challenges and overall cost would be alleviated if consensus conferences were institutionalized and convened on a regular basis by an experienced team. We also hope that institutionalization of such a process would have the added benefit of increasing channels of communication with relevant policymakers. Unlike in Denmark, where consensus conferences are convened by the Danish Board of Technology, a government agency that advises Danish Parliament on science and technology issues, the United States does not have the structure or precedent for communicating a lay panel’s findings with policy makers. We were happy with our efforts to convey the panel’s recommendations to federal and state agencies considering biomonitoring, and to a broader audience of scientists and advocates, but believe the project may have had even more impact on policy makers if a more formal relationship existed. Lay panel members invested a tremendous amount of time and energy into the process, and if they doubt their findings are given consideration, we worry this could lead to skepticism about participating in future consensus conferences or similar projects.

## Conclusion

The consensus conference process demonstrated that the public can make informed recommendations on complex scientific and technical information. During three weekends, panelists developed their own expertise and took knowledge back to their communities. They posed insightful questions to experts and issued thoughtful responses to public questions. They were engaged with the issue, grasped the full range of uncertainties and complexities, and formulated reasoned and useful recommendations.

The deliberations and results of the Boston Consensus Conference on Human Biomonitoring demonstrate that the consensus conference process is effective in educating and engaging a small but diverse cross section of the public in scientific questions, one goal of research translation. It shows that the public is fully capable of understanding technical issues and making valuable contributions to discussions about policy-relevant science and technology. More important, however, the process was effective at bringing perspectives of the public to bear on scientific research and public health practice, an important element of simultaneous research translation. The lay panel’s unique perspective and recommendations on biomonitoring surveillance, communication, and ethics are important for environmental health scientists and all stakeholders involved in biomonitoring. Indeed, they have very practical implications for recruitment of and communication with participants in biomonitoring studies, and for the establishment of the many state-level biomonitoring surveillance programs now being considered.

Research translation should be a reciprocally informative process that allows for mutual education, with products that are strengthened by the diversity of voices and perspectives that create them. We hope the success of this attempt to engage lay people on a complicated technical topic and gather their input will encourage others to use this model for public participation in science and technology policy making.
